# Hybrid Multi-Access Method for Space-Based IoT: Adaptive Bandwidth Allocation and Beam Layout Based on User Distribution

**DOI:** 10.3390/s24186082

**Published:** 2024-09-20

**Authors:** Qingquan Liu, Lihu Chen, Songting Li, Yiran Xiang

**Affiliations:** College of Aerospace Science and Engineering, National University of Defense Technology, Changsha 410073, China; liuqq_wy2022@163.com (Q.L.); songtingl@sina.com (S.L.); xiang_yr@sina.com (Y.X.)

**Keywords:** beam layout, multiple access, user distribution, user capacity, space-based Internet of Things

## Abstract

The development of space-based Internet of Things is limited by insufficient allocable frequency resources and low spectrum utilization. To meet the demand for massive access users under the condition of restricted frequency resources, a multi-dimensional hybrid multiple-access method for space-time-frequency-code division based on user distribution (MHSTFC-UD) is established. It divides the beam cell of a low orbit satellite into the central and edge area and dynamically adjusts the radius of the central area and the allocation of frequency resources according to the distribution of users. The optimization model for the radius of the central area and the allocation of frequency resources is established and solved by the genetic algorithm. Also, it takes the edge area as the protection interval to realize the full-frequency multiplexing between the beam cells in the time domain, space domain and code domain. The simulation results show that compared with the traditional method of frequency reuse in two or three dimensions, the multi-dimensional hybrid multiple-access method can improve the maximum access capacity of a single satellite user by one to three orders of magnitude. Moreover, the MHSTFC-UD can increase users by an additional 11.5% to 33.1% compared to fixed area division and frequency resource allocation.

## 1. Introduction

The space-based Internet of Things (S-IoT) is a network that uses various space-based platforms to acquire, process, transmit and apply Internet of Things (IoT) information [1]. It connects various devices and sensors through a satellite network to enable large-scale connection and data transmission between devices and sensors on a global scale [2]. It has the characteristics of working in all weather conditions and across all regions and is not easily disrupted [3,4]. Nowadays, it is widely used in the ship automatic identification system (AIS), automatic dependent surveillance-broadcast (ADS-B), and data collection system (DCS) [5]. The S-IoT not only makes up for the shortcomings of ground-based IoT [6] but also combines with ground-based IoT, which is one step further from realizing the interconnection of everything. However, the further development of S-IoT is being limited by insufficient allocable frequency resources and low spectrum utilization [7].

With the rapid development of mobile communications and IoT, the number of terrestrial terminals and users showed explosive growth. Global System for Mobile Communication Association (GSMA) and Transforma Insights predict that the global scale of IoT connections in 2030 will reach 30 billion [8] and access to satellite terminals will reach more than 100 million users. The demand for spectrum resources is growing. The Ku band, as the “golden band” for LEO satellite communication, has almost been allocated. The remaining LEO satellite communication bands that can be allocated are Ka and V bands. Although the communication band is higher and the available band is wider, the rain fade of Ka and V communication bands is also larger, and the requirements for the receiver are also higher. The remaining available spectrum resources in the L, S and C frequency bands, where meteorological and maritime communication satellites work, are limited. The existing technologies can no longer meet the increasing communication needs.

The low utilization of an allocated spectrum is another constraint for the development of space-based IoT. According to a survey conducted by the Federal Communications Commission (FCC) in the United States, the resource utilization rate of the licensed frequency bands is between 15% and 85% under the preset spectrum allocation model [9]. Moreover, in Finland, more than 90% of the spectrum is idle [10]. Therefore, under the condition of limited frequency resources, improving the utilization of spectrum resources by combining different multiple-access technologies, optimizing spectrum allocation, and frequency multiplexing will be the key to expanding the system capacity and the development of satellite communication technology [7].

To improve the utilization of a spectrum, the combination of multiple-access techniques can achieve frequency multiplexing. Ref. [11] combines code division multiple access (CDMA) with space division multiple access (SDMA), but the access user capacity is more significantly affected by the spreading sequence length and the size of the spreading gain. Then, the K-means clustering algorithm is used to cluster and group users to each SDMA group to improve throughput [12]. Ref. [13] proposes a MIMO transmission scheme for full-frequency multiplexing (FFR), but it needs to know the channel information of the users for precoding. Further, a single-frequency multi-beam transmission (SF-MBT) algorithm allocates different beams more carefully for different geographic regions to avoid the interference generated by full frequency multiplexing [14].

Therefore, improving the beam layout is also one of the ways to improve spectrum utilization, such as concentric space division multiplexing (CSDM) based on orbital angular momentum (OAM) [15], dividing the beam into central and edge regions [16,17], designing multicolor frequency multiplexing layouts [18,19], and designing square coverage layouts by using polygonal splitting strategies [20]. However, most are only used in terrestrial networks. Ref. [21] proposes combining SDMA with precoding to improve a system’s capacity by reducing interference between users, while as the density of user distribution increases, more and more accurate channel state information (CSI) needs to be provided.

In practical application scenarios, user distribution is uneven and random. Therefore, a dynamic allocation of beam bandwidth resources according to the user channel state [22,23] and a beam-hopping algorithm (BH) based on service requirements are proposed [24,25,26,27]. Although the resource allocation is time- and space-varying, it still cannot meet the needs of an exponential increase in the number of users. However, the combination of dynamically allocated resources and pre-emptive channels will lead to packet loss due to message squeezing [28]. In addition, ref. [29] proposes an adaptive grouping strategy based on the user density under each beam coverage to optimize the beam layout for ADS-B signal reception, providing s a way to adjust the beam layout based on the user distribution.

This article mainly focuses on the study of the S-IoT using narrowband satellites to collect IoT information from various sensors. When faced with the demand for IoT terminal user access at the level of hundreds of millions of users under the condition of limited frequency resources, it is necessary to maximize spectrum utilization and reduce frequency idle time. However, most of the aforementioned research work is focused on satellite signal downlinking, and most of the solutions with high spectrum utilization adopt fixed resource allocations. This results in a situation where a few users occupy most of the frequency resources. The overall spectrum utilization of solutions based on users dynamically adjusting resources is low and cannot meet the needs of massive user access. Therefore, for uplink signals, we propose to improve the frequency reuse rate from the four dimensions of space, time, frequency, and code, and dynamically adjust the frequency resource allocation within the beam and the beam layout according to the user distribution in order to increase the user capacity of the S-IoT. The main contributions of this paper are summarized as follows:

- To increase the user capacity of the S-IoT, a multi-dimensional hybrid multiple-access method for space-time-frequency-code division based on user distribution (MHSTFC-UD) is proposed. It divides a single beam cell into a central and edge region, with the edge region serving as a protection interval. Combining TDMA, FDMA, and CDMA, full-frequency multiplexing is possible between beams, which can increase the number of users by one to three orders of magnitude.
- To improve spectrum utilization, we propose dynamically adjusting the allocation of frequency resources and the beam layout based on the user distribution. By adjusting the radius of the central area and the frequency allocation ratio between the central area and the edge area, the situation where allocated frequency resources are not used can be reduced.
- We propose to use the genetic algorithm to optimize the radius of the central area and the proportion of frequency resources allocated. Compared with fixed resource allocation and layout methods, the MHSTFC-UD can increase user access by about 27.5%.

The rest of this paper is organized as follows. Section 2 introduces the MHSTFC-UD model and the simulation of the terrestrial user distribution model. Section 3 introduces the beam layout and frequency allocation optimization algorithm based on user distribution and takes the user access amount as the optimization target. Section 4 conducts simulation comparison experiments. Finally, Section 5 summarizes the research results of this paper.

## 2. System Model

### 2.1. The Model of the Multi-Dimensional Hybrid Multiple-Access Method for Space-Time-Frequency-Code Division

Ground user terminals of the S-IoT are in a dormant state most of the time to reduce energy consumption and prolong their service life. Therefore, when a satellite receives a message from the ground terminal, it first sends a wake-up command to the ground terminal and then allocates a channel for the user.

**Figure 1:**
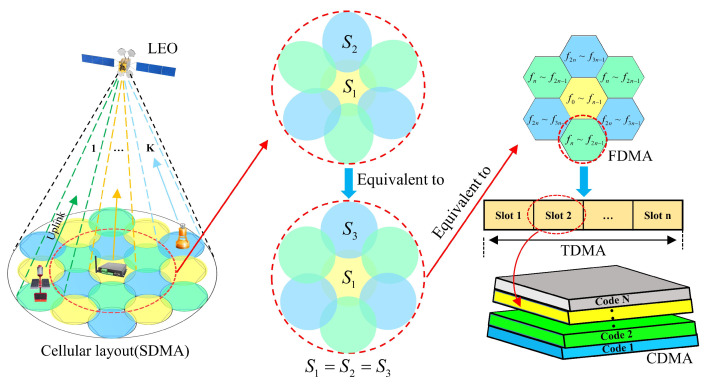
Simplified model of a LEO satellite’s cellular beam layout.

The S-IoT is based on narrowband communication. Thus, we establish a MH-STDF model under the condition of limited frequency resources. It includes a single satellite and several different beams, with the sub-beams arranged in a cellular pattern, as shown in Figure 1. The LEO satellite cellular beam layout can be regarded as consisting of multiple small cells. Except for the circular coverage area of the main beam, the coverage areas of the other beams are elliptical. For the ease of analysis, the elliptical coverage areas are proportionally equivalent to circular coverage areas of equal area. Furthermore, the shape of the coverage areas is equivalent to a regular hexagon. The user distribution within the coverage areas is also equivalent proportional. The coverage areas of beams are multiplexed using three colors. Within a single beam cell, frequency division multiple access (FDMA), time division multiple access (TDMA), and CDMA are used to further multiplex the frequencies within the beam. In this model, when the satellite sends a wake-up command, it assigns information such as the frequency point, time slot, and code pattern to the terminal.

In order to increase the maximum number of users that can be connected to a single satellite, each beam cell needs to allocate as much frequency resources as possible to improve the overall spectrum utilization. Therefore, in this model, the beam cells are divided into central and edge areas according to the distance from the center of the sub-beam. The edge area is used as a protection interval to avoid user channel interference in the overlapping area between adjacent beams. The central area allocates more frequency resources according to user needs as much as possible.

The frequency allocation principle for a single satellite multi-beam cell is as follows: First, the available frequency is divided into multiple sub-bands according to FDMA. Then, the frequency band resources are allocated to the central and edge areas of the cell. It is ensured that the frequency bands allocated to the central area do not overlap with those allocated to the edge area in the same beam. The edge area serves as a protection interval, and the frequency band resources allocated to adjacent edge areas cannot overlap. The allocation of frequency resources to users in the same region is random to ensure that all users can get services fairly.

The principle of time slot allocation is as follows: Since the LEO satellite beam is fast-moving, it faces the problem of beam switching [30,31]. Therefore, when dividing time slots, some channels are reserved for users not currently under the beam. The remaining channels are allocated to users close to the edge of the beam according to their location. The time slot allocation of users among different satellite beams can be transmitted via the intersatellite link [32,33].

**Figure 2:**
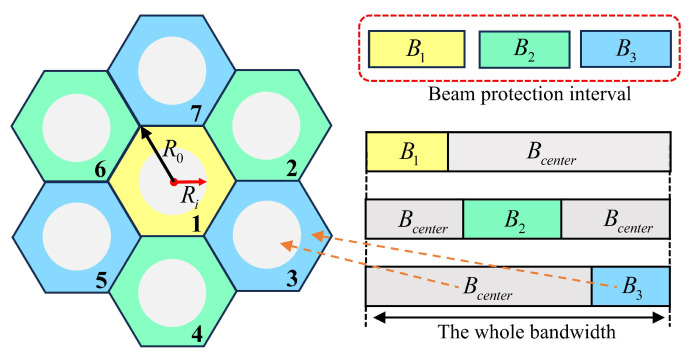
Frequency allocation of the MHSTFC-UD model.

The frequency resource allocation of the MHSTFC-UD model is shown in Figure 2. Full-frequency multiplexing is implemented in different beams, and three-color multiplexing is implemented between adjacent edge cells to avoid frequency interference.

After a single beam cell has divided the time slot through organized time division multiple access (OTDMA) and allocated a certain amount of frequency resources, the model further implements frequency reuse by using PN codes through CDMA to expand the spectrum, thereby further increasing the number of users that can be accessed to the satellite.

The MHSTFC-UD model distinguishes different access users in four dimensions: space, time, frequency and code. If the user is different in any dimension, the signal can be easily separated. Therefore, the uplink signal received by the antenna pointing to the central area on the LEO satellite is the user’s desired signal from the central area and the weak interference signal of the same frequency from the adjacent beam cell. The signal received by the antenna pointing to the edge area on the LEO satellite is the user’s desired signal from the edge area and the weak interference signal of the same frequency from the central area of the adjacent beam cell.

### 2.2. User Distribution Model within the Beam Coverage Area

Due to the high orbital position, the LEO satellite has a beam coverage area that is much larger than that of ground-based stations. The characteristics of the distribution of ground user terminals are different in different regions. When the subsatellite point changes, the distribution characteristics of the ground terminals in the corresponding coverage area also change. Also, the number of users in each sub-beam is also constantly changing.

**Figure 3:**
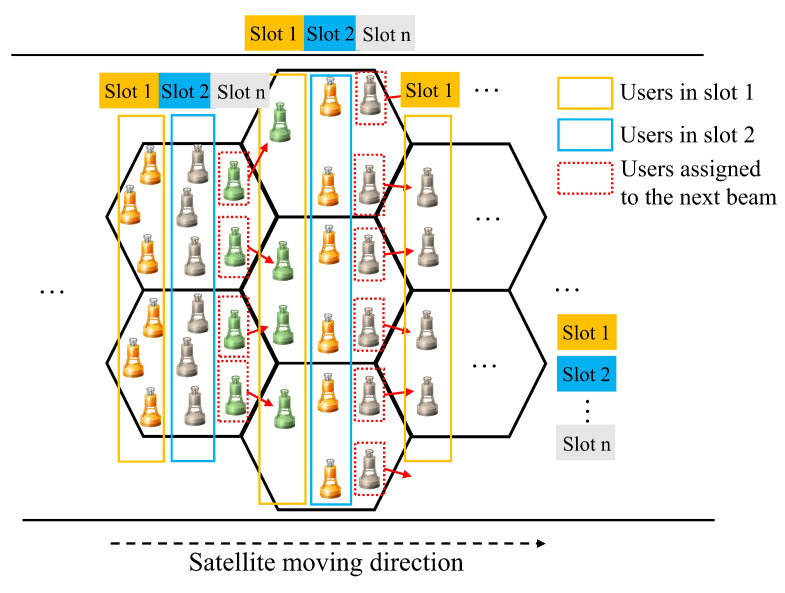
User division in sub-beam cell.

Therefore, the actual number of users within a single sub-beam cell is jointly determined by the location of the user terminal, the time slot division, and the satellite motion state, as shown in Figure 3. Meanwhile, the sub-beam coverage areas partially overlap with each other, and the short message transmission duration is in the millisecond range. When dividing the area of users belonging to the overlapping area of sub-beam coverage, it can be divided according to whether their transmission message time will exceed the current beam coverage area. Furthermore, the total number of users in the beam cell within a period of time can be determined through OTDMA. The number is certain and can provide a basis for frequency allocation.

The LEO satellite moves much faster than the ground mobile sensor terminal, so the low-orbit satellite can be considered stationary. Both fixed and moving terminals move relative to the satellite. In the case of multiple short messages sent by a single ground terminal, it can be considered as multiple users with the same initial position sending a single short message in different time slots.

**Figure 4:**
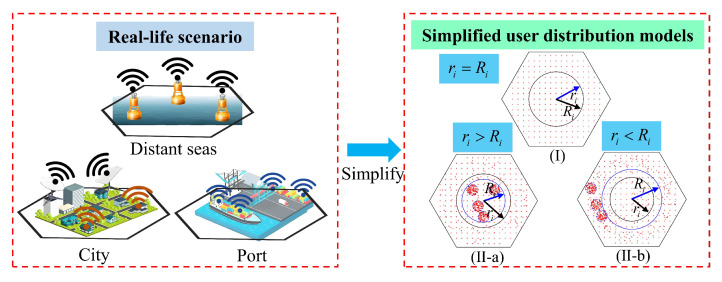
Ground user distribution model: (**I**) Users are uniformly distributed. (**II-a**) Users are randomly distributed, and the density of user distribution is higher within $r_{i}$. (**II-b**) Users are randomly distributed, and the density of user distribution is higher outside $r_{i}$.

So, we establish the following two models for the distribution of ground users. And three different radius are defined. $R_{0}$ is the sub-beam coverage radius and $R_{i}$ is the central area radius of frequency band resource allocation, as shown in Figure 2. $r_{i}$ is the central area of user distribution, as shown in Figure 4.

- Scenario 1: Sensor terminals located in remote areas or on the distant seas can be approximately equivalent to users evenly distributed in the same beam, with different numbers of users in different beams, $\rho_{i}=\rho_{i}^{\prime }$, as shown in Figure 4I.
- Scenario 2: Users are randomly and densely distributed within the beam. For example, a large number of ground sensor terminals are deployed in densely populated areas such as cities and ports [34]. The distribution of user terminals is divided equally into users concentrated inside or outside a region with a radius of $r_{i}$, $p_{i}>q_{i}\;\;\;\;$ or $\;\;\;\;p_{i}<q_{i},\;\;\;$*i* = [1, *n*] and $0<r_{i}<R_{0}$, as shown in Figure 4(II-a,II-b).

The detailed notations and definitions are summarized in Table 1.

## 3. Beam Layout and Frequency Allocation Optimization Method Based on User Distribution

To meet the explosive growth in the number of users accessing the S-IoT, we optimize the frequency resources allocated to the central and edge areas and frequency assignment center area radius $R_{i}$ through the genetic algorithm. When the number of users requesting access on the ground far exceeds the maximum number of satellite accesses, it can obtain the maximum number of user accesses as much as possible.

### 3.1. Number of Users in the Coverage Area for a Satellite

The user distribution under the satellite beam, as shown in Figure 3, can be equivalently expressed as independent user densities $p_{i}$ and $q_{i}$ for the center and edge regions, where $p_{i}$ corresponds to the user density within a circular area with radius $r_{i}$. The number of users $U_{i}$ in the *i*th beam can then be expressed as

(1) $$U_{i}=U_{c}^{i}+U_{s}^{i}=p_{i}\pi r_{i}^{2}+q_{i}\pi \left(R_{0}^{2}-r_{i}^{2}\right)$$

The total number of users wait for access to the satellite $U_{total}$ is

(2) $$U_{total}=\sum_{i=1}^{K}U_{i}=\sum_{i=1}^{K}p_{i}\pi r_{i}^{2}+q_{i}\pi \left(R_{0}^{2}-r_{i}^{2}\right)$$

In the *i*th beam, when $0<R_{i}\leq R_{0}$, the relationship between the average distribution density of users in the center of the beam $\rho_{i}$ and frequency assignment center area radius $R_{i}$ is

(3) $$\rho_{i}=\left\{\begin{array}{c} p_{i},R_{i}\leq r_{i}\\ \left(p_{i}\pi r_{i}^{2}+q_{i}\pi \left(R_{i}^{2}-r_{i}^{2}\right)\right)/\pi R_{i}^{2},R_{i}>r_{i} \end{array}\right.$$

### 3.2. Number of Actual Accessible Users for a Satellite

The distribution of users in different beams is different, and so is the number of users. To improve spectrum utilization, the frequency resource allocation between beams and within and outside the same beam needs to be dynamically adjusted according to the actual distribution of users.

The number of users that can actually be accessed depends on the user channel. During the uplink process, the channel gain $h_{i}^{m}$ for a single user is

(4) $$h_{i}^{m}=G_{t}\cdot G_{r}\cdot PL$$

(5) $$PL={\left(\frac{c}{4\pi d_{i}f_{c}}\right)}^{2}$$

where $G_{t}$ and $G_{r}$ represent the signal transmission gain and reception gain, respectively; PL is the loss of the signal path from the user terminal to the satellite; $d_{i}$ is the distance between the user terminal and the satellite signal; $f_{c}$ is the carrier frequency of the signal.

The received signal power $P_{i}^{m}$ of a single user on the satellite is

(6) $$P_{i}^{m}=P_{0}^{m}\cdot h_{i}^{m}=P_{0}^{m}\cdot G_{t}\cdot G_{r}\cdot PL$$

To measure the channel conflict that may be caused by frequency multiplexing in the MHSTFC-UD model, the conflict factor α is defined.

The reasons that may cause channel interference are the frequency resources allocated to adjacent beams overlap under the same time slot and code sequence, and the code sequence repeats within the same beam under the same time slot and frequency.

(7) $$\alpha_{i}=\frac{B_{i}\cap B_{j}}{B_{i}}+\frac{\cap Code_{m}}{\sum_{m=1}^{U_{i}}Code_{m}}-\frac{B_{i}\cap B_{j}}{B_{i}}\cdot \frac{\cap Code_{m}}{\sum_{m=1}^{U_{i}}Code_{m}}$$

(8) $$B_{i}=B_{s}^{i}+B_{c}^{i}$$

$B_{s}^{i}$ and $B_{c}^{i}$ represent the bandwidth allocated to the edge area and the center area within the same beam, respectively; $Code_{i}$ represents different code patterns.

The user distribution position under the satellite beam is different, and the path loss caused by the near and far effect is also different. To avoid excessive signal power of some terminals and interference to other signals during demodulation, the power of users in the beam cell is controlled so that the signal strength reaching the receiving end is equal. Then, the SINR of the beam cell is

(9) $$SINR_{i}=\frac{\sum_{m=1}^{U_{i}}P_{i}^{m}}{N_{0}B_{i}+\sum_{m=1}^{U_{i}}\alpha_{i}P_{i}^{m}}$$

Therefore, the throughput $C_{i}$ of the *i*th beam in a unit time slot is

(10) $$C_{i}=C_{s}^{i}+C_{c}^{i}=B_{i}\log_{2}\left(1+SINR_{i}\right)$$

To further increase the user capacity, TDMA and CDMA are used. For CDMA, it is

(11) $$N_{t}=\frac{\left(M-1\right)P_{i}^{m}}{B_{w}}+N_{0}$$

(12) $$E_{b}=\frac{P_{i}^{m}}{V_{code}}$$

Furthermore, the signal-to-noise ratio $\frac{E_{b}}{N_{t}}$ is

(13) $$\frac{E_{b}}{N_{t}}=\left(\frac{B_{w}}{V_{code}}\right)\cdot \frac{P_{i}^{m}}{N_{0}B_{w}+\left(M-1\right)P_{i}^{m}}$$

That is, the number of users can grow, as follows:

(14) $$M=\frac{B_{w}}{R}\cdot \frac{N_{t}}{E_{b}}-\frac{N_{0}B_{w}}{P_{i}^{m}}+1$$

For TDMA, according to the satellite transit time *T* and the time required for a single message $\frac{L}{V_{t}}$, the number of time slots that can be divided $N_{slot}$ can be obtained as follows:

(15) $$N_{slot}=\frac{TV_{t}}{L}$$

Therefore, when the throughput required by a single user is $C_{0}=B_{m}\log_{2}\left(1+SINR_{m}\right)$, the total number of users $N_{user}$ that can be accessed to a single satellite is

(16) $$C_{i}=M\cdot N_{slot}\cdot B_{i}\log_{2}\left(1+SINR_{i}\right)$$

(17) $$N_{user}=\sum_{i=1}^{K}C_{i}/C_{0}$$

### 3.3. Optimization of Beam Layout and Frequency Allocation

The number of users that can actually be connected to the satellite $N_{sum}$ is also related to the number of users currently distributed under different beams; thus,

(18) $$N_{sum}=\sum_{i=1}^{K}\left[\min\left\{\begin{array}{cc} C_{c}^{i}/C_{0} & \rho_{i}\pi R_{i}^{2} \end{array}\right\}+\min\left\{\begin{array}{cc} C_{s}^{i}/C_{0} & U_{i}-\rho_{i}\pi R_{i}^{2} \end{array}\right\}\right]$$

Define η to facilitate the representation of the adjacent location relationship between the beams.

(19) $$\eta_{i}^{j}=\left\{\begin{array}{c} \begin{array}{cc} 1 & ,if\;B_{c}^{i}\;\;\;\;is\;\;\;\;adjacent\;\;\;\;to\;\;\;\;B_{s}^{j} \end{array}\\ \begin{array}{cc} 0 & ,if\;B_{c}^{i}\;\;\;\;is\;\;\;\;\;not\;\;\;adjacent\;\;\;\;to\;\;\;\;B_{s}^{j} \end{array} \end{array}\right.\;\;\;\;$$

By adjusting the frequency resource allocation ratio of the central area and the edge area $B_{s}^{i},B_{c}^{i}$ and the frequency resource allocation center area radius $R_{i}$, the number of users connected to a single satellite is the optimization target. The complete optimization problem is

(20) $$\left\{\begin{array}{c} Object:\max\left(N_{sum}\right)\\ s.t.\;\;\;\;C1:0<B_{s}^{i}+B_{c}^{i}\leq B,\forall B_{s}^{i}\subset B,B_{c}^{i}\subset B\\ C2:\sum_{i}^{K-2}B_{s}^{i}+\eta_{i}^{i+1}B_{s}^{i+1}+\eta_{i}^{i+2}B_{s}^{i+2}\leq B_{0}\\ C3:0<R_{i}<R_{0},i=1,2,...,n \end{array}\right.$$

where constraint C1 indicates that the frequency band resources allocated to the central and edge areas of the same beam cannot overlap and cannot exceed the total amount of frequency resources that can be allocated; constraint C2 indicates that the frequency band resources allocated to adjacent edge areas cannot overlap; and constraint C3 indicates that the central area cannot exceed the sub-beam.

In the above optimization problem, the bandwidth allocated to the edge area $B_{s}^{i}$ of each beam cell and the frequency resource allocation center area radius $R_{i}$ are both variables to be optimized. There are 19 beam cells in total, and the variables are independent of each other. In addition, the objective function in Equation (20) is nonlinear and can be solved on the star. Therefore, we use the genetic algorithm to solve for the maximum number of access users. The specific parameter settings are shown in Table 2, and the steps are as follows.

- Randomly initialize the frequency resource allocation center area radius and the frequency resources allocation in the central and edge areas of the 19 beams.
- Calculate the value of the objective function for the initial population.
- Individual selection, chromosomal crossing and genetic variation are performed on the population to generate a new population of offspring.
- Determine whether the number of iterations has been reached. If so, the process is complete and the optimal solution has been found. Otherwise, repeat the above steps.

## 4. Simulation Results and Performance Analysis

In this paper, the orbital parameters of the FM13 satellite in the ORBCOMM constellation of the S-IoT are selected for simulation, and the user access capacity of the MHSTFC-UD under the condition of limited frequency resources is compared with the fixed resource allocation of MHSTFC, UFFR [17] and BH [26] methods. The specific simulation parameter settings are shown in Table 3.

### 4.1. Throughput Compared to Traditional Methods

**Figure 5:**
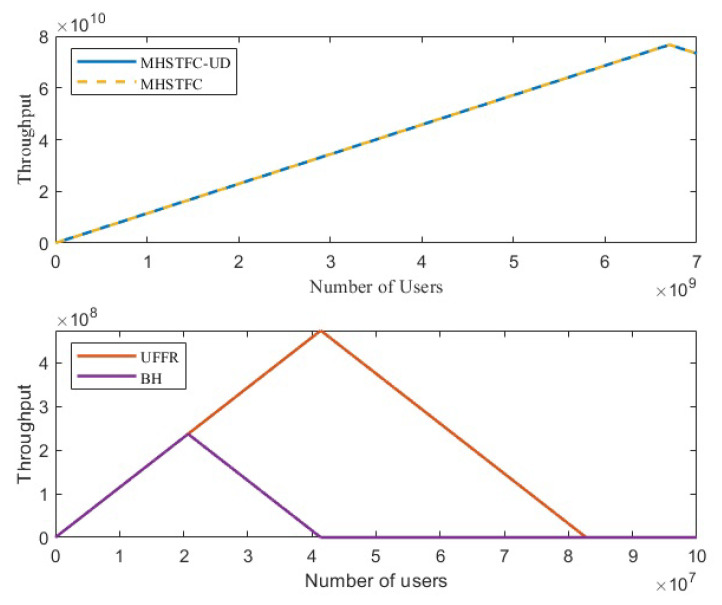
System throughput as a function of the number of users with a uniform user distribution.

To analyze the change in throughput, we simulate the MHSTFC-UD model when the number of users increases under the ideal scenario of uniform distribution of users within the beam and compare its throughput with the traditional scheme. User distribution density can fully utilize all frequency resources. It is also assumed that each user only sends a short message once during the satellite’s one-time pass. The simulation results are shown in Figure 5 and the maximum throughput is shown in Table 4.

The MHSTFC-UD model distinguishes between different users in four dimensions, time, space, frequency and code, compared to the UFFR [17] and BH [26] methods, which distinguish between different users only in the time or space domain or a combination of the two. Therefore, its throughput can reach 76.6 Gbps when the number of users reaches 6.69 billion. Compared with UFFR and BH, the maximum throughput is increased by about 162 times and 319 times, respectively. However, in practice, the distribution of users is random, so this throughput is often not achieved.

Therefore, in the next section, the system’s access performance is analyzed when the users are randomly distributed according to the actual situation. Moreover, to more intuitively illustrate the number of users accessing the system, the throughput is converted into the number of users accessing the system for comparative analysis.

### 4.2. Number of Users Accessing the System in a Non-Uniform Scenario

**Figure 6:**
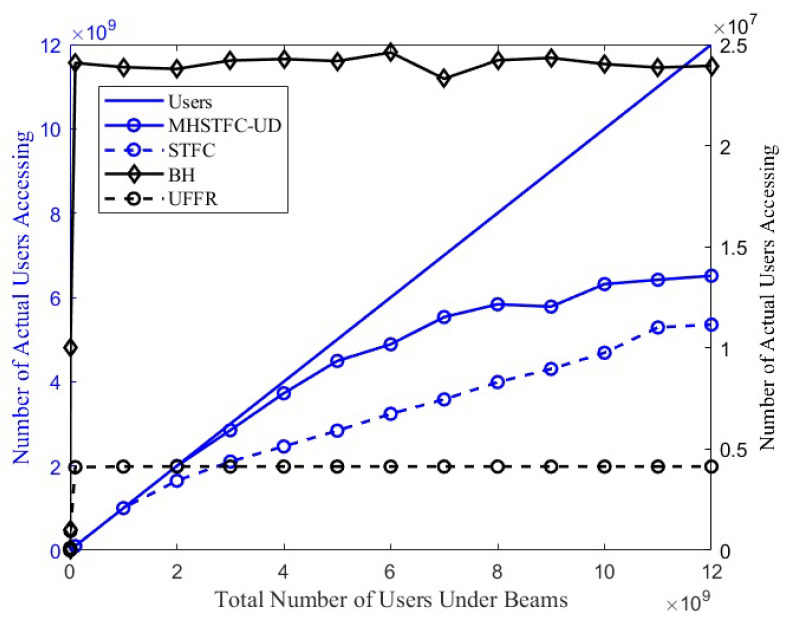
The number of actual users accessing the system changes with the total number of users under the beam.

Under the condition of limited frequency resources, the actual number of users accessing the system is an important indicator for measuring different methods when faced with the massive user access demand that exceeds the upper limit of access. In this section, we simulate the actual user access volume of the MHSTFC-UD as the number of users changes. It is assumed that users are randomly distributed within the beam and that each user only sends a short message once during the satellite’s one-time pass. The simulation results are shown in Figure 6 and the maximum user capacity is shown in Table 5.

As the number of users under the satellite beam increases from 0 to the order of a billion, the BH and UFFR reach the upper threshold limit before the MHSTFC-UD. Using the MHSTFC-UD, the access needs of all users can be met when the number of users does not exceed $2\times 10^{9}$, and it is not affected by the user distribution. When the number of users exceeds $2\times 10^{9}$, the MHSTFC-UD cannot meet all user access needs, and the maximum number of user connections that can be accommodated is $5.8\times 10^{9}$. The degree of fluctuation of the maximum number of users that MHSTFC-UD can access is affected by the user distribution. However, compared with the traditional scheme, the MHSTFC-UD uses a scheme that jointly distinguishes different users from four dimensions, which can increase the number of connected users by about 241 to 1585 times.

Next, the actual user access volume under the MHSTFC-UD and under the MHSTFC with fixed-band resource allocation and fixed center and edge area division is compared.

**Figure 7:**
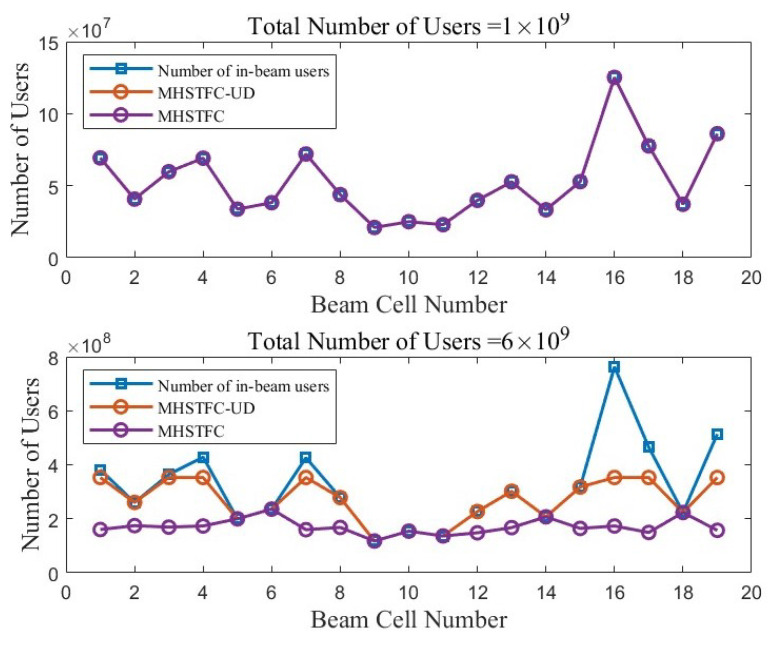
The number of actual users accessing to the system changes with the total number of users under the beam.

Figure 7 shows a comparison of the MHSTFC-UD and MHSTFC when the number of users in the beam does not exceed $2\times 10^{9}$ and both can meet the access needs of all users. The number of users that can access each sub-beam is the same. When the number of users exceeds $2\times 10^{9}$, both cannot meet the access needs of all users. However, the MHSTFC-UD can dynamically adjust resource allocation based on the user distribution and can meet the access needs of more users than the MHSTFC. Therefore, when the total number of users is $6\times 10^{9}$, the MHSTFC-UD can meet the access needs of about 27.5% more users than the MHSTFC.

## 5. Conclusions

In this paper, to improve the access number of ground terminal users of the S-IoT under the condition of limited frequency resources, the MHSTFC-UD is established. Users are distinguished in the four dimensions of space, time, frequency, and code. The central area and edge area are divided in each sub-beam, and the edge area is used as a protection interval. By dynamically adjusting resource allocation and beam layout, the number of user accesses is increased by 241 to 1585 times compared to the traditional method. Moreover, compared with fixed resource allocation, the dynamic resource adjustment scheme based on the user distribution can increase user access by about 11.5% to 33.1%. However, when users need to send multiple short or long messages during the time when the satellite passes overhead, they will occupy the channels of other users, resulting in a certain reduction in the user access of this model. Overall, compared with the traditional method, user access can still be increased by one to two orders of magnitude.

## Figures and Tables

**Table 1:** Notations and definitions.

Notations	Definitions
*K*	Number of beams
$N_{slot}$	Number of time slots
*L*	Message length
$V_{t}$	Transmission rate
$V_{code}$	Code rate
*T*	Satellite ground coverage time
$U_{c}^{i}$	Number of users in the center of the *i*th beam
$U_{s}^{i}$	Number of users in the edge area of the *i*th beam
*B*	Bandwidth
$B_{c}^{i}$	Bandwidth resources allocated to the center area of the *i*th beam
$B_{s}^{i}$	Bandwidth resources allocated to the edge area of the *i*th beam
$B_{w}$	Bandwidth after spread spectrum
$B_{m}$	Bandwidth per user
$C_{i}$	User throughput of the *i*th beam
$C_{s}^{i}$	User throughput in the edge area of the *i*th beam
$C_{c}^{i}$	User throughput in the center of the *i*th beam
$R_{0}$	Beam radius
$r_{i}$	Radius from the center of a sub-beam in the *i*th beam
$R_{i}$	Radius of the central area of the frequency allocation of the *i*th beam
$p_{i}$	User distribution density in the *i*th beam within $r_{i}$
$q_{i}$	User distribution density in the *i*th beam out $r_{i}$
$N_{i}$	Number of users that can be connected to the *i*th beam
$N_{user}$	Maximum number of users that can be connected to a single satellite
$N_{sum}$	Maximum number of users actually connected to a single satellite
PL	Path loss
$P_{i}$	User terminal signal transmission power
$G_{r}$	Receive gain
$G_{t}$	Transmit gain
$N_{0}$	Noise power spectral density

**Table 2:** Parameter settings for the genetic algorithm.

Parameters	Value
Number of Variables	38
Crossover Fraction	0.8
Generations	3800
Population Size	200
Migration Fraction	0.2
Migration Interval	20

**Table 3:** Simulation parameters.

Parameters	Value
Orbit semi-major axis *a*/km	7157.21
Orbit inclination i/deg	45.055
Signal frequency *f*/GHz	1
Number of beams *K*	19
Beam radius $R_{0}$/km	209.66
Bandwidth *B*/MHz	10
Message length *L*/Byte	32
Transmit rate $V_{t}$/bps	2400
Bandwidth per user $B_{m}$/Hz	4200

**Table 4:** Comparison of maximum user throughput in the case of an even user distribution.

Method	MHSTFC-UD	MHSTFC	UFFR	BH
Maximum throughput	76.6 Gbps	76.6 Gbps	0.47 Gbps	0.24 Gbps
Multiplication factor	319.17	319.17	1.96	1

**Table 5:** Comparison of maximum user throughput in the case of an even user distribution.

Method	MHSTFC-UD	MHSTFC	UFFR	BH
Upper threshold for full user access	$2\times 10^{9}$	$1\times 10^{9}$	$4\times 10^{6}$	$2\times 10^{7}$
Maximum user capacity	$6.5\times 10^{9}$	$5.4\times 10^{9}$	$4.1\times 10^{6}$	$2.5\times 10^{7}$
Multiplication factor in maximum user capacity	1585.37	1317.07	1	6.10

## Data Availability

The data presented in this study are available upon request from the corresponding author.
